# Experimental Evaluation of Balance Prediction Models for Sit-to-Stand Movement in the Sagittal Plane

**DOI:** 10.1155/2013/592328

**Published:** 2013-09-26

**Authors:** Oscar David Pena Cabra, Takashi Watanabe

**Affiliations:** ^1^Graduate School of Engineering, Tohoku University, Aramaki-aza-Aoba 6-6-11-901-7, Aoba-ku, Sendai 980-8579, Japan; ^2^Graduate School of Biomedical Engineering, Tohoku University, Aramaki-aza-Aoba 6-6-11-901-7, Aoba-ku, Sendai 980-8579, Japan

## Abstract

Evaluation of balance control ability would become important in the rehabilitation training. In this paper, in order to make clear usefulness and limitation of a traditional simple inverted pendulum model in balance prediction in sit-to-stand movements, the traditional simple model was compared to an inertia (rotational radius) variable inverted pendulum model including multiple-joint influence in the balance predictions. The predictions were tested upon experimentation with six healthy subjects. The evaluation showed that the multiple-joint influence model is more accurate in predicting balance under demanding sit-to-stand conditions. On the other hand, the evaluation also showed that the traditionally used simple inverted pendulum model is still reliable in predicting balance during sit-to-stand movement under non-demanding (normal) condition. Especially, the simple model was shown to be effective for sit-to-stand movements with low center of mass velocity at the seat-off. Moreover, almost all trajectories under the normal condition seemed to follow the same control strategy, in which the subjects used extra energy than the minimum one necessary for standing up. This suggests that the safety considerations come first than the energy efficiency considerations during a sit to stand, since the most energy efficient trajectory is close to the backward fall boundary.

## 1. Introduction

Lower limb motor functions are important for the activities of daily living (ADL), participating in social activities, and preventing bedridden state. Therefore, rehabilitation training of sit-to-stand movement is considered to be the first step to prevent motor-disabled patients and elderly people from being bedridden. In the rehabilitation, joint angle trajectories and/or joint torques are commonly measured for evaluation of motor function. During lower limb movement, however, balance control is also required for developing the movement safely. Since sit-to-stand movement requires control of stability in addition to muscular strength [1, 2], balance control ability in sit-to-stand movement should also be evaluated in rehabilitation training.

Bipedal balance has been studied since it is expected to have important applications in preventing health problems associated with falls [3–6] and in designing better and safer rehabilitation techniques for lower limb function impairment [7]. Bipedal dynamic stability has also been studied as the ability to restore static balance [8, 9]. In those studies, it has been theoretically shown that the ability to restore balance is described by conditions of the center of mass (CM) velocity-position with respect to the base of support (BOS). 

In order to calculate these BOS-CM conditions for predicting the ability to control gait balance, those previous studies modeled human bipedal gait as a single joint, simple inverted pendulum [3, 6, 8, 9]. Based on the method using the simple inverted pendulum model, dynamic balance during sit-to-stand movement has also been studied [10–12]. However, sit-to-stand movements have multiple joint interactions that present significant variation of the CM rotational radius of the inverted pendulum model. In spite of the aspect of sit-to-stand movements, it has not been studied sufficiently if a simple inverted pendulum would suffice to describe the balance in the sit-to-stand movements or not.

In this paper, in order to make clear usefulness and limitation of the traditional simple inverted pendulum model in balance prediction in sit-to-stand movements, the balance prediction obtained by the method of using the traditional simple model was compared to the prediction of a complex model that included multiple-joint influence. Here, since a telescopic pendulum model has been shown to be no less informative than more demanding multisegment models [13], an inertia variable inverted pendulum model, in which rotational radius was varied during sit-to-stand movement, was used as the complex model. The model predictions were tested upon experimentation by measuring sit-to-stands performed by healthy subjects. In addition, normal sit-to-stand CM trajectories were discussed from the measured data for evaluating balance control ability. 

## 2. Methods

### 2.1. Outline of Balance Prediction

In the sit-to-stand movement, the center of mass (CM) velocity-position is in a given initial state when the subject leaves the chair. Then, the task of sit-to-stand consists of stopping the CM somewhere over the base of support (BOS) while satisfying the restrictions imposed by the friction coefficient, the foot geometry, maximum and minimum physiological ankle torque, and the condition that the foot segment should not move.

**Figure 1:**
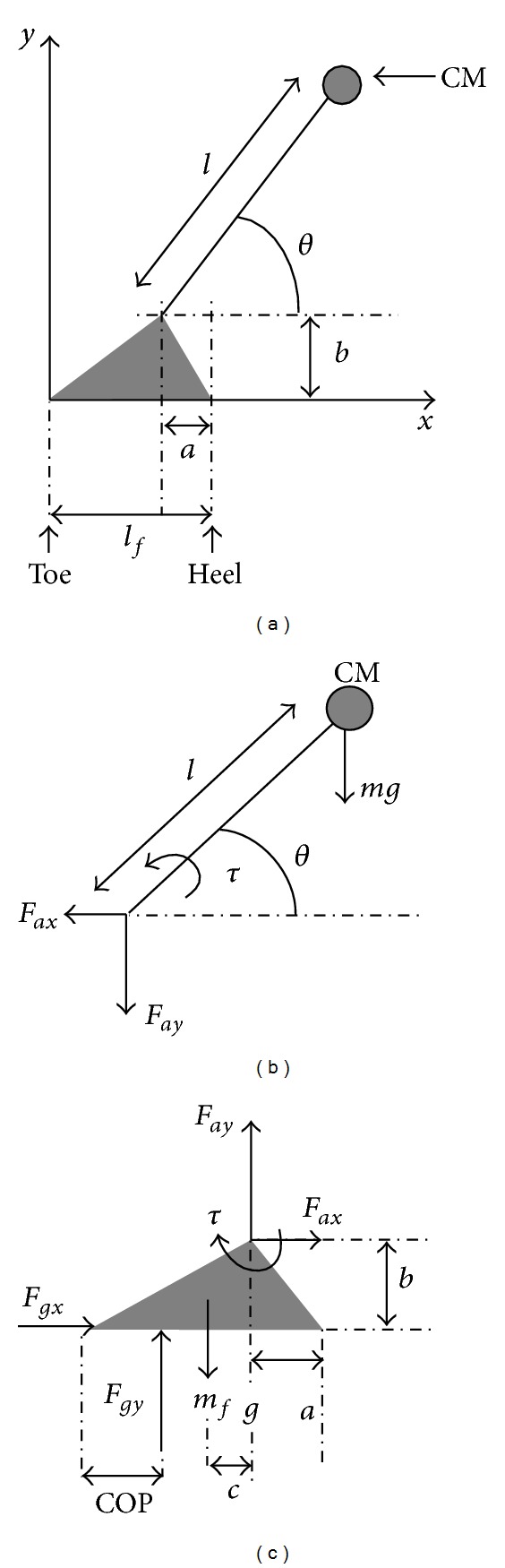
The inverted pendulum used for modeling sit-to-stand movement in the sagittal plane. Note that the length *l* is constant for the traditional simple inverted pendulum model and is represented by a function of the angle *θ* in order to include multiple-joint influence for the complex model used in this paper.

**Figure 2:**
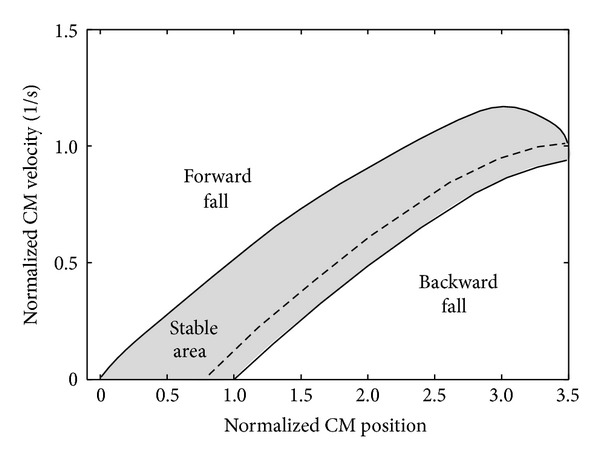
An example of the dynamic balance condition obtained through a simple inverted pendulum. The horizontal axis represents the posterior position of the CM, measured with respect to the toe and normalized to the subject's foot length (thus the 0 and 1 represent the toe and heel position, resp.). The vertical axis represents the anterior velocity of the CM normalized to the subject's height. The solid lines enclose the CM velocity-position conditions that allow recovering static balance (gray-shaded area), that is, the dynamic balance conditions. The broken line represents the most energy-efficient trajectory (zero external torque).

The sit-to-stand balance control feasibility can be calculated by finding all the CM velocity-position conditions when leaving the chair that allow the CM to arrive and stop over the base of support using the inverted pendulum model shown in Figure 1 with the maximum ankle plantar flexion and the maximum ankle dorsiflexion. The results were plotted in the form of a map as shown in Figure 2, in which the solid lines are the theoretical balance control boundaries. The upper boundary is the trajectory where the subject manages to stop the CM just over the toe using maximum plantar flexion, and therefore velocity-position conditions over the top boundary would result in a forward fall. The lower boundary is the trajectory where the subject manages to take the CM just over the heel using maximum dorsiflexion, and velocity-position conditions below the bottom boundary would result in a backward fall. The broken line shows the conditions that would allow recovering static balance without using ankle torque after the seat-off.

### 2.2. Models

The inverted pendulum model with a static support segment (Figure 1) has a physical state that can be described with two variables, the angle and the angular velocity. If the joint torque is known, the behavior of the pendulum is completely described. However, in order to maintain the conditions of no support segment movement, this torque is subjected to several physical constraints. Re-writing every torque constraint as a function of the state variables, it is possible to find the torque ranges for every possible state of the system (see appendix for details). These torque ranges will be the allowed control ranges and therefore can be used to simulate the pendulum movement and to measure the controllability of the system. The models used in this paper for balance prediction in sit-to-stand movement are described below (see appendix for details). 

#### 2.2.1. Simple Inverted Pendulum Model

In previous studies, a simple inverted pendulum model with constant pendulum length *l* was used in Figure 1 for predicting the ability to control gait balance, since it had no redundancy and only one degree of freedom. A biped can be modeled by a support segment (foot) that does not move and the rest of the body that rotates around the ankle. Previous studies on dynamic balance during sit-to-stand movement also used the simple inverted pendulum model [10–12]. The total external torque measured with respect to the ankle *τ* is equal to the time variation of the angular momentum of the body. This was described by the following [8]:

(1) $$\begin{array}{c} \sum \tau =\frac{d}{dt}\left(I\dot{\theta }\right). \end{array}$$

That is,

(2) $$\begin{array}{c} \tau -mgl\cos\theta =ml^{2}\ddot{\theta }, \end{array}$$

where *I*, *θ*, *m*, *g*, and *l* stand for the CM rotational inertia, the angle of rotation of the CM, the body mass, the gravitational acceleration, and the pendulum length, respectively.

#### 2.2.2. Inertia (Rotational Radius) Variable Inverted Pendulum Model

In this paper, the methodology presented by Pai and Patton [8] was expanded for predicting balance in sit-to-stand movement. Since the CM rotational radius of the inverted pendulum model (*l* in Figure 1) varies significantly in the sit-to-stand movement due to multiple joint interactions, a telescopic inverted pendulum model [13] that was shown to be useful to represent movements including multiple joint interactions was used. Here, variation of rotation radius of the pendulum was represented by variation of the CM rotational inertia *I*(*t*). Therefore, the total external torque measured with respect to the ankle *τ* can be written as the following equation:

(3) $$\begin{array}{c} \sum \tau =\frac{d}{dt}\left(I\dot{\theta }\right)=\frac{dI}{dt}\dot{\theta }+I\frac{d\dot{\theta }}{dt}. \end{array}$$

Assuming that the different joints of the body move synchronized for a given movement, it is possible to define the rotational inertia of the body as a function of the rotational angle of the CM *I*(*θ*). This assumption leads to the following equation:

(4) $$\begin{array}{c} \tau -mgl\cos\;\theta =\frac{dI\left(\theta \right)}{d\theta }{\dot{\theta }}^{2}+I\left(\theta \right)\ddot{\theta }. \end{array}$$

By defining the total body inertia as only a function of the rotational angle, the pendulum model reduces to a one degree of freedom system. Thus, it becomes possible to use the same methodology developed by Pai and Patton [8] to predict balance in the sit-to-stand movement. 

It is important to mention that the inclusion of the inertia (rotational radius) variation affects not only the rotational movement equation (3), but also all the constraints equations (refer to the appendix).

### 2.3. Experimental Methods

Six healthy male subjects (25.3 ± 7.7 years old) participated in measurements of sit-to-stand movements for determination of their inertia function and for evaluation of the balance predictions. The CM velocity-position was estimated by measuring the position of the head, trunk, thigh, shank, and foot segments and estimating their mass distribution from the subject's weight [14]. The arm position was not used because a preliminary experiment performed in our study showed that it did not have much influence on the CM position calculation. It was assumed that the mass of each segment was uniformly distributed in each of the segments, and therefore the CM of a given body segment would be at the center of each of the segments. The body CM can be finally calculated from the position of the CM of all the segments. 

Experimental data were recorded using 15 reflective markers with an 8-camera, 3D motion analysis system at a data sampling rate of 120 Hz (Vicon, Oxford Metrics, UK). Force plates were used for finding the timing of seat-off. All the theoretical balance predictions were calculated using MATLAB (Math Works, Inc., USA).

First, every subject was asked to perform two self-selected most natural sit-to-stands, to measure their sit-to-stand inertia function. The rotational inertia function was estimated by assigning the measured CM rotational radius (*l*(*θ*)) to the rotational angle (*θ*):

(5) $$\begin{array}{c} I\left(\theta \right)=ml{\left(\theta \right)}^{2}, \end{array}$$

(6) $$\begin{array}{c} l\left(\theta \right)=a_{0}\theta^{3}+a_{1}\theta^{2}+a_{3}\theta +a_{4}. \end{array}$$

Here, *a*_0_~*a*_4_ are parameters to approximate measured CM rotational radius. These functions were used to calculate the balance predictions during the sit-to-stand by the complex model that included multiple-joint influence.

Next, the subjects were asked to stand up at different initial conditions of the CM position which in turn will lead to different initial conditions of the CM velocity-position when the subject leaves the chair. In order to create different velocity-position CM conditions at the seat-off, the horizontal position of the feet was varied while sitting. The feet were shifted −0.2~1.2 foot lengths forward from the foot position where the ankle was at 90 degree, since those distances were showed to include successful and not successful stand ups. Every subject was asked to perform a total of 23 to 25 sit-to-stands. It is important to note that the actual position of the feet is rather unimportant since the real important information will be the horizontal BOS-CM distance that is precisely known from the markers position measurements.

## 3. Results

The BOS-CM horizontal position was gradually increased to demanding standing up conditions for every subject. A threshold of the BOS-CM horizontal distance of the first unsuccessful sit-to-stand was set to divide the data into two groups. The first group would be the conditions where every subject was able to make a successful sit-to-stand (normal condition). The second group would be all the data after the threshold (demanding condition), which was used for evaluating the theoretical balance predictions since it would include unstable sit-to-stand movements. From the results, the sitting BOS-CM horizontal distance threshold was 2.48 foot length. A total of 146 sit-to-stand measurements were performed, but due to markers disappearances or subjects' mistakes (BOS movement), 127 valid sit-to-stands were analyzed, in which 80 sit-to-stands were classified as the normal condition and 47 sit-to-stands as the demanding one.

**Figure 3:**
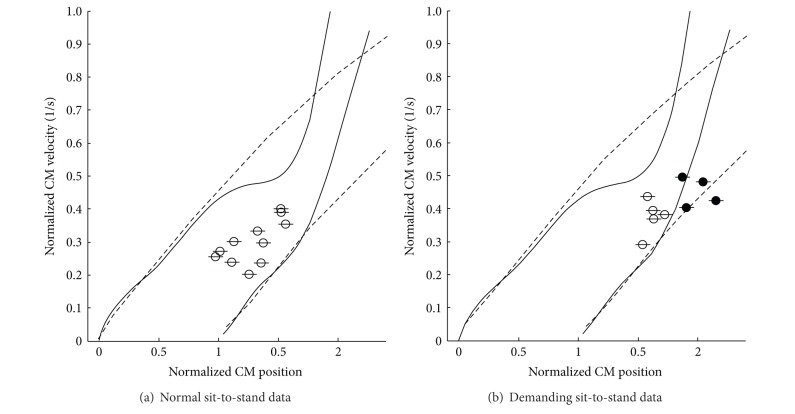
An example of balance control prediction for normal sit-to-stand conditions (a) and demanding sit-to-stand conditions (b) (subject C). The solid lines show the boundaries for sit-to-stand balance control calculated with the inertia variable model, while the broken lines show the boundaries calculated with a simple inverted pendulum model. The open circles represent the successful sit-to-stand data while the closed circles represent the unsuccessful sit-to-stand data. In order to consider a data to be stable, the whole error bar on the point should be inside the boundaries.

Figure 3 shows an example of the balance control predictions calculated for one of the subjects. The plots on the maps show the CM velocity-position conditions of the measured sit-to-stands at the seat-off. The horizontal line in every data plot shows the error of the CM position estimation (±1.5 cm), which was calculated from the standard deviation of the mass distribution of the biomechanical model used to estimate the mass of each segment of subjects [14]. Data were considered to be stable only if the whole error bar was inside the boundaries. The results for all the subjects were that both the simple inverted pendulum model method and the inertia variable model method correctly classified all the 80 normal sit-to-stands as seen in Figure 3(a). As for the 47 demanding sit-to-stands, the complex model including multiple-joint influence showed a stability sensitivity of 100% and a specificity of 82.4% in the balance prediction. For the same data, the simple inverted pendulum prediction showed a sensitivity of 100%, but a specificity of 41.2%.

**Figure 4:**
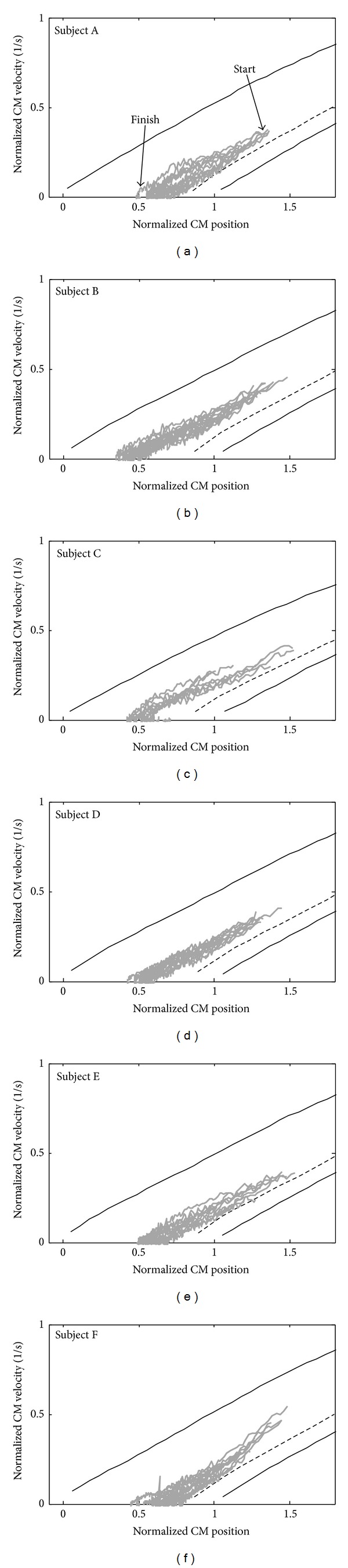
Measured CM sit-to-stand trajectories after leaving chair. Here, the 72 trajectories that showed a more energetic (faster) CM trajectory than the most efficient zero torque trajectory (in broken lines) are shown.

**Figure 5:**
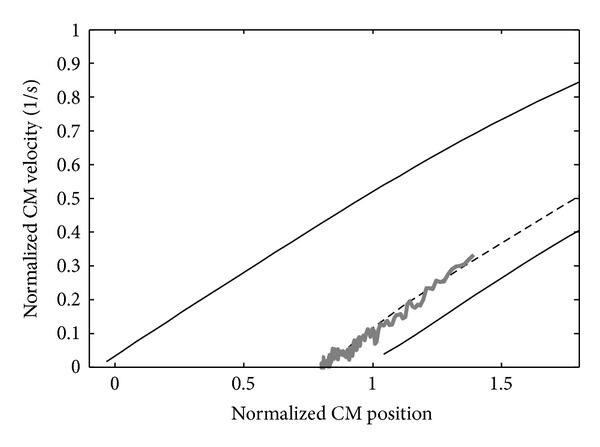
An example of a nonstandard control strategy performed by subject A. The CM position at the seat-off was 1.39 foot length. It is possible to see that at the moment of the seat-off, the CM is in a backward position near the most efficient zero torque compared to the trajectories of the same subject in Figure 4.

From the 80 normal sit-to-stands measured, it was found that 72 (90%) of them had extra kinetic energy compared to the (calculated) zero torque trajectory, while the other 8 data were almost on their zero torque trajectories crossing the zero torque condition after leaving chair.  Figure 4 shows all of the 72 trajectories divided by the subject and plotted over their corresponding stability map. The broken line shows the zero torque trajectory which would be the most energy-efficient trajectory to make a successful sit-to-stand [8]. The gray lines show the measured CM sit-to-stand trajectories. On the other hand, only a few data (8 measured trajectories) did cross the zero torque condition.  Figure 5 shows an example of this CM behavior during a sit-to-stand performed by subject A.

## 4. Discussion

**Figure 6:**
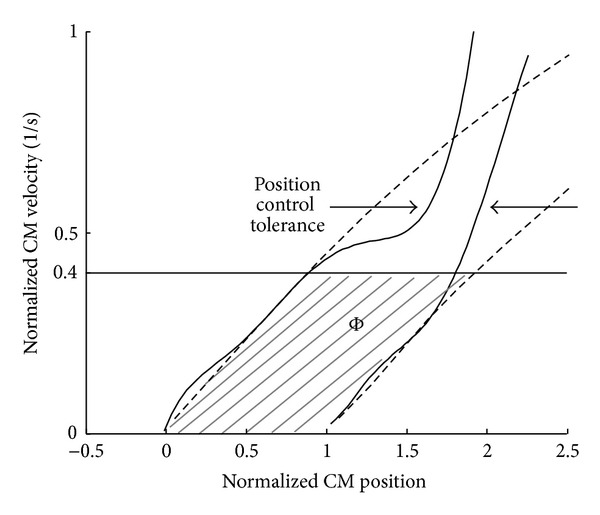
An example of validity threshold for the simple inverted pendulum model. The plot on the map shows average velocity at the seat-off for a natural sit-to-stand.

The results from the demanding sit-to-stand conditions showed that the inertia variable pendulum model is better than a simple inverted pendulum model for evaluating the stability of a sit-to-stand movement. The specificity for the demanding sit-to-stand was improved from 41% to 82% by using the model including multiple-joint influences. On the other hand, both of the maps correctly classified all the normal (natural) sit-to-stand data as seen in Figure 3(a). Here, a similarity threshold between the maps was defined as the value that was calculated as the difference of the position control tolerance (the horizontal distance of the boundaries of the maps) becomes less than a certain value. For example, by setting the similarity threshold of 85%, it was found that the simple inverted pendulum prediction for sit-to-stands showed similar results as the inertia variable pendulum model up to CM velocities of 0.4 heights per second (see Figure 6). The CM velocity of 0.4 heights per second is much higher than the average velocity of the normal sit-to-stand data (0.24 ± 0.03 heights per second) measured with healthy subjects in this paper. Therefore, the simple inverted pendulum model can be valid for normal sit-to-stand movements.

The simple inverted pendulum model is also considered to be reliable for rehabilitation assessment and balance analysis in sit-to-stand movements of motor-disabled subjects and elderly persons. For sit-to stand movements that have low CM velocity at the seat-off, there was no large difference in the map between the simple inverted pendulum model and the inertia variable pendulum model as shown in Figure 6. In the studies on dynamic balance during sit-to-stand movement, CM velocities at the seat-off of elderly persons were similar to or lower than young subjects [11] and those of persons with Parkinson disease were lower than those of healthy elderly subjects [12]. These suggest that normal sit-to-stand movements of motor-disabled subjects and elderly persons show lower CM velocities than those of natural (normal) sit-to-stand of healthy young subjects, which could be evaluated by the simple inverted pendulum model.

As seen in Figure 4, the CM control strategy in a normal sit-to-stand condition is different from the most energy-efficient strategy shown by the broken lines. For a given CM position, it is possible to see that the CM velocity is higher than the velocity required for achieving a successful sit-to-stand. This extra velocity would come from an extra effort before the subject leaves the chair and then would require an extra effort to absorb it after the subject leaves the chair. These suggest that the natural sit-to-stand control strategy has a velocity-position target that satisfies very stable physical conditions even if that means an extra energy cost. These can also be understood when comparing the CM trajectories and the balance control boundaries in Figure 4. It is clear that this energy inefficiency allows a greater balance control tolerance, improving the stability against a dangerous backward fall (lower boundary). 

It seems that the natural gait control strategy first tries to satisfy the stability requirements rather than optimizing the energy usage. However, energy considerations are often used when analyzing gait and designing assistive and rehabilitation technology [15, 16]. It is suggested that not only energy but also stability may play a major role in control strategy and therefore should be also taken into consideration. 

As for the data that crossed the zero torque line as shown in Figure 5, it was found that most of them had a backward CM position at the seat-off. During the measurements, the subjects were asked to stand up in different CM conditions including very backward positioned CM conditions. It is considered that the subjects did not use the standard control strategy or they tried but failed to achieve their intended forward velocity when they were standing up in different foot positions than usual.

## 5. Conclusions

The balance prediction in sit-to-stand movements obtained using a traditional simple inverted pendulum was compared to the prediction of an inertia variable inverted pendulum model including multiple-joint influence. The results showed that the multiple-joint influence model is more accurate in predicting balance during sit-to-stand movements under demanding conditions and also that the traditionally used simple inverted pendulum is still reliable in predicting balance during normal or nondemanding sit-to-stand movements. Especially, the simple inverted pendulum model could be effective for sit-to-stand movements with low CM velocity at the seat-off. In addition, almost all CM trajectories during normal sit-to-stands seemed to follow the same control strategy, in which the subjects used extra energy than the minimum one necessary for standing up. This suggests that the safety considerations come first before the energy-efficiency considerations during a sit-to-stand since the most energy efficient trajectory is close to the backward fall boundary.

## Appendix

Equations (A.1)–(A.6) describe the motion of the pendulum and the BOS (see Figure 1). Notice that the inertia variation is introduced making the pendulum length a function of the angle (see (3)–(5))

(A.1) $$\begin{array}{c} \sum \tau =\frac{d}{dt}\left(I\dot{\theta }\right)=\frac{dI}{dt}\dot{\theta }+I\frac{d\dot{\theta }}{dt}\left(\text{CM}\right),\\ \tau -mgl\cos\theta =\;\frac{dI}{d\theta }{\dot{\theta }}^{2}+I\ddot{\theta }=2ml\frac{dl}{d\theta }{\dot{\theta }}^{2}+ml^{2}\ddot{\theta },\\ \ddot{\theta }=\frac{\tau -mgl\cos\theta -2ml\left(dl/d\theta \right){\dot{\theta }}^{2}}{ml^{2}}=\frac{\tau -mlJ}{ml^{2}}. \end{array}$$

Here, $J=g\cos\theta +2\left(dl/d\theta \right){\dot{\theta }}^{2}$.

For the CM,

(A.2) $$\begin{array}{c} -F_{ax}=m{\ddot{x}}_{\text{CM}}, \end{array}$$

(A.3) $$\begin{array}{c} -F_{ay}-mg=m{\ddot{y}}_{\text{CM}}. \end{array}$$

For the foot,

(A.4)

(A.5)

(A.6)

Transforming the constraints shown by (A.7)–(A.10) into restrictions of the ankle torque would make it possible to find the feasible torque for the simulation

(A.7) $$\begin{array}{c} F_{gy}\geq 0, \end{array}$$

(A.8) $$\begin{array}{c} \left|F_{gx}\right|\leq \mu F_{gy}, \end{array}$$

(A.9) $$\begin{array}{c} 0<\text{COP}<l_{f}, \end{array}$$

(A.10) $$\begin{array}{c} \tau_{\text{maximum}\;\text{Plantarflexion}}<\tau <\tau_{\text{maximum}\;\text{Dorsiflexion}}. \end{array}$$

From the constraint of the ground reaction force (A.7) and (A.3) and (A.6),

(A.11) $$\begin{array}{c} m{\ddot{y}}_{\text{CM}}+\left(m_{f}+m\right)g\geq 0,\\ {\ddot{y}}_{\text{CM}}+\left(\frac{m_{f}}{m}+1\right)g\geq 0,\\ \left(\ddot{\theta }A_{1}+{\dot{\theta }}^{2}B_{1}\right)+\left(\frac{m_{f}}{m}+1\right)g\geq 0,\\ \left(\ddot{\theta }A_{1}+{\dot{\theta }}^{2}B_{1}\right)+K_{1}\geq 0, \end{array}$$

where,

(A.12) $$\begin{array}{c} A_{1}=\frac{dl}{d\theta }\sin\theta +l\cos\theta ,\\ B_{1}=\frac{d^{2}l}{d\theta^{2}}\sin\theta +2\frac{dl}{d\theta }\cos\theta -l\sin\theta ,\\ K_{1}=\left(\frac{m_{f}}{m}+1\right)g. \end{array}$$

From (A.1), it becomes

(A.13) $$\begin{array}{c} \left(\tau -mlJ\right)A_{1}\geq -ml^{2}\left({\dot{\theta }}^{2}B_{1}+K_{1}\right). \end{array}$$

Then, solving for *τ*

(A.14) $$\begin{array}{c} \tau \geq -\frac{ml^{2}}{\left|A_{1}\right|}\left({\dot{\theta }}^{2}B_{1}+K_{1}\right)+mlJ,\;\text{for}\;A_{1}>0,\\ \tau \leq -\frac{ml^{2}}{\left|A_{1}\right|}\left({\dot{\theta }}^{2}B_{1}+K_{1}\right)+mlJ,\;\text{for}\;A_{1}<0. \end{array}$$

Equation (A.8) gives the friction anterior and friction posterior constraints. For the maximum anterior friction condition, (A.8) becomes

(A.15) $$\begin{array}{c} F_{gx}\geq -\mu F_{gy}. \end{array}$$

From (A.2), (A.5) and (A.3), (A.6),

(A.16) $$\begin{array}{c} m{\ddot{x}}_{\text{CM}}\geq -\mu \left\{m{\ddot{y}}_{\text{CM}}+\left(m_{f}+m\right)g\right\},\\ {\ddot{x}}_{\text{CM}}\geq -\mu {\ddot{y}}_{\text{CM}}-\left(\frac{m_{f}}{m}+1\right)g\mu ,\\ \ddot{\theta }A_{x}+{\dot{\theta }}^{2}B_{x}\geq -\mu \left(\ddot{\theta }A_{1}+{\dot{\theta }}^{2}B_{1}\right)+K_{2}, \end{array}$$

where,

(A.17) $$\begin{array}{c} A_{x}=\frac{dl}{d\theta }\cos\theta -l\sin\theta ,\\ B_{x}=\frac{d^{2}l}{d\theta^{2}}\cos\theta -2\frac{dl}{d\theta }\sin\theta -l\cos\theta ,\\ K_{2}=-\mu K_{1}. \end{array}$$

Then,

(A.18) $$\begin{array}{c} \ddot{\theta }A_{2}\geq {\dot{\theta }}^{2}B_{2}+K_{2}, \end{array}$$

where,

(A.19) $$\begin{array}{c} A_{2}=\mu A_{1}+A_{x},\\ B_{2}=-\mu B_{1}-B_{x}. \end{array}$$

From (A.1),

(A.20) $$\begin{array}{c} \left(\tau -mlJ\right)A_{2}\geq ml^{2}\left({\dot{\theta }}^{2}B_{2}+K_{2}\right). \end{array}$$

Solving for *τ*,

(A.21) $$\begin{array}{c} \tau \geq \frac{ml^{2}}{\left|A_{2}\right|}\left({\dot{\theta }}^{2}B_{2}+K_{2}\right)+mlJ,\;\text{for}\;\;A_{2}>0,\\ \tau \leq \frac{ml^{2}}{\left|A_{2}\right|}\left({\dot{\theta }}^{2}B_{2}+K_{2}\right)+mlJ,\;\text{for}\;\;A_{2}<0. \end{array}$$

For the maximum posterior friction condition, (A.8) becomes

(A.22) $$\begin{array}{c} F_{gx}\leq \mu F_{gy}. \end{array}$$

From (A.2), (A.5) and (A.3), (A.6),

(A.23) $$\begin{array}{c} m{\ddot{x}}_{\text{CM}}\leq \mu \left\{m{\ddot{y}}_{\text{CM}}+\left(m_{f}+m\right)g\right\},\\ {\ddot{x}}_{\text{CM}}\leq \mu {\ddot{y}}_{\text{CM}}+\left(\frac{m_{f}}{m}+1\right)g\mu ,\\ \ddot{\theta }A_{3}\leq {\dot{\theta }}^{2}B_{3}+K_{3}, \end{array}$$

where,

(A.24) $$\begin{array}{c} A_{3}=A_{x}-\mu A_{1}=A_{2}-2\mu A_{1},\\ B_{3}=\mu B_{1}-B_{x}=B_{2}+2\mu B_{1},\\ K_{3}=\mu K_{1}=-K_{2}; \end{array}$$

from (A.1)

(A.25) $$\begin{array}{c} \left(\tau -mlJ\right)A_{3}\leq ml^{2}\left({\dot{\theta }}^{2}B_{3}+K_{3}\right); \end{array}$$

solving for *τ*

(A.26) $$\begin{array}{c} \tau \leq \frac{ml^{2}}{\left|A_{3}\right|}\left({\dot{\theta }}^{2}B_{3}+K_{3}\right)+mlJ,\;\text{for}\;\;A_{3}>0,\\ \tau \geq \frac{ml^{2}}{\left|A_{3}\right|}\left({\dot{\theta }}^{2}B_{3}+K_{3}\right)+mlJ,\;\text{for}\;\;A_{3}<0. \end{array}$$

Equation (A.9) gives the COP heel and COP toe constraints. For the COP behind the heel condition, (A.9) becomes

(A.27) $$\begin{array}{c} \text{COP}<l_{f}. \end{array}$$

Equation (A.4) becomes

(A.28) $$\begin{array}{c} l_{f}\geq l_{f}-\left(\frac{bF_{gx}-\tau +cm_{f}g}{F_{gy}}+a\right),\\ 0\geq -aF_{gy}-bF_{gx}+\tau -cm_{f}g. \end{array}$$

From (A.2), (A.5) and (A.3), (A.6),

(A.29) 0≥(−a){my¨CM+(mf+m)g} −b(mx¨CM)+τ−cmfg,−amy¨CM−bmx¨CM+τ≤a(mf+m)g+cmfg−am(A1θ¨+B1θ˙2)−bm(Axθ¨+Bxθ˙2)+τ≤K4θ¨E1+τ+θ˙2D1≤K4,

where,

(A.30) E1=−amA1−bm(A2−μA1),D1=−amB1+bm(B2+μB1),K4=a(mf+m)g+cmfg.

From (A.1),

(A.31) $$\begin{array}{c} \left(\tau -mlJ\right)E_{1}+\tau ml^{2}\leq ml^{2}\left(K_{4}-{\dot{\theta }}^{2}D_{1}\right); \end{array}$$

solving for *τ*

(A.32) $$\begin{array}{c} \tau \left(ml^{2}+E_{1}\right)\leq ml^{2}\left(K_{4}-{\dot{\theta }}^{2}D_{1}\right)+mlJE_{1},\\ \tau \leq \frac{ml^{2}\left(K_{4}-{\dot{\theta }}^{2}D_{1}\right)+mlJE_{1}}{\left|ml^{2}+E_{1}\right|},\;\;\text{for}\;ml^{2}+E_{1}>0,\\ \tau \geq \frac{ml^{2}\left(K_{4}-{\dot{\theta }}^{2}D_{1}\right)+mlJE_{1}}{\left|ml^{2}+E_{1}\right|},\;\text{for}\;ml^{2}+E_{1}<0. \end{array}$$

For the COP after the toe condition, (A.9) becomes

(A.33) $$\begin{array}{c} 0<\text{COP}. \end{array}$$

Equation (A.4) becomes

(A.34) $$\begin{array}{c} 0<l_{f}-\left(\frac{bF_{gx}-\tau +cm_{f}g}{F_{gy}}+a\right),\\ 0<\left(l_{f}-a\right)F_{gy}-bF_{gx}+\tau -cm_{f}g. \end{array}$$

From (A.2), (A.5) and (A.3), (A.6),

(A.35) 0<(lf−a)(my¨CM+(mf+m)g) −b(mx¨CM)+τ−cmfg,(lf−a)my¨CM−bmx¨CM+τ  ≥−(lf−a)(mf+m)g+cmfg,m(lf−a)(A1θ¨+B1θ˙2)−mb(Axθ¨+Bxθ˙2)+τ≥K5θ¨E2+τ+θ˙2D2≥K5,

where,

(A.36) E2=m(lf−a)A1−mb(A2−μA1),D2=m(lf−a)B1+mb(B2+μB1),K5=−(lf−a)(mf+m)g+cmfg.

From (A.1)

(A.37) $$\begin{array}{c} \left(\tau -mlJ\right)E_{2}+\tau ml^{2}+\geq ml^{2}\left(K_{5}-{\dot{\theta }}^{2}D_{2}\right). \end{array}$$

Solving for *τ*,

(A.38) $$\begin{array}{c} \tau \left(ml^{2}+E_{2}\right)\geq ml^{2}\left(K_{5}-{\dot{\theta }}^{2}D_{2}\right)+mlJE_{2},\\ \tau \geq \frac{ml^{2}\left(K_{5}-{\dot{\theta }}^{2}D_{2}\right)+mlJE_{2}}{\left|ml^{2}+E_{2}\right|},\;\text{for}\;ml^{2}+E_{2}>0,\\ \tau \leq \frac{ml^{2}\left(K_{5}-{\dot{\theta }}^{2}D_{2}\right)+mlJE_{2}}{\left|ml^{2}+E_{2}\right|},\;\text{for}\;ml^{2}+E_{2}<0. \end{array}$$
